# Pertussis detection in children with cough of any duration

**DOI:** 10.1186/s12887-019-1615-3

**Published:** 2019-07-12

**Authors:** Dan-Xia Wu, Qiang Chen, Kai-Hu Yao, Lan Li, Wei Shi, Jiang-Wei Ke, Ai-Min Wu, Peng Huang, Kun-Ling Shen

**Affiliations:** 1 Beijing Children’s Hospital Affiliated to Capital Medical University, No. 56 Nan-Li-Shi Road, Beijing, 100045 China; 2 Department of Respiratory Medicine, Jiangxi Provincial Children’s Hospital, Nanchang, China; 3 Beijing Institute of Pediatrics, Beijing Children’s Hospital, Beijing, China; 4 Department of Clinical Laboratory, Jiangxi Provincial Children’s Hospital, Nanchang, China

**Keywords:** Whooping cough, Children, Prevalence, Characteristics

## Abstract

**Background:**

The diagnosis of pertussis in clinical practice continues to be a challenge worldwide as the symptoms are variable. We aimed to determine the prevalence of pertussis in Chinese children irrespective of cough duration and explore the clinical characteristics of children with pertussis with different cough durations.

**Methods:**

This was a prospective study of children 1 month to 11 years of age with different cough durations in one large Chinese hospital. Bilateral deep posterior nasopharyngeal swabs and venepuncture for full blood count, CRP and serology and sputum were obtained when possible for investigation. E-test strips were used for testing the susceptibility of the *B.pertussis* isolates against erythromycin, azithromycin, sulphamethoxazole/trimethoprim, levofloxacin, amoxicillin and doxycycline. Demographic, clinical and laboratory information on culture and antimicrobial susceptibility testing was collected from children, and analyzed using SAS v.10 (SAS Institute Inc., USA).

**Results:**

After exclusions we analyzed 312 children. Ninety-seven (31.1%) children had laboratory evidence of pertussis. When grouped by cough duration, few characteristics were significant between children with and without pertussis. Of the 36 isolates, 72.2% (26/36)could not be inhibited by erythromycin and azithromycin at all. The MIC50 and MIC90 to amoxicillin were 0.75 mg/L and 1 mg/L respectively, sensitive to amoxicillin by the EUCAST points.

**Conclusions:**

The “one-size-fits-all” clinical pertussis case definition is no longer optimal to recognize this disease. A large comprehensive study of children with all types of cough is required to make substantial inroads into increasing both the sensitivity and specificity in pertussis diagnosis, which will have a beneficial impact on public health. Amoxicillin maybe an alternative for children with marolide-resistant *B.pertussis* infection; however, local sensitivities are required to inform clinical practice.

## Background

The diagnosis of pertussis, or whooping cough, in clinical practice continues to be a challenge worldwide as the symptoms are variable [1]. Pragmatically, it is an overlooked cause of cough in children after several decades of universal immunization [2]. The symptoms and its subsequent clinical diagnosis are influenced by age and presence of underlying co-morbidities such as rhinovirus, respiratory syncytial virus (RSV), adenovirus, *Streptococcus pneumoniae*, *Mycoplasma pneumoniae* and *Staphylococcus aureus*, etc. [3, 4]. In addition, immunization status or history of natural infection, presence of passively acquired antibody, and antibiotic treatment may also have a role [3].

Pertussis is caused by the fastidious Gram-negative bacterium *Bordetella pertussis* [5]. It is estimated that there are 30 to 50 million cases of pertussis worldwide every year, leading to more than 300,000 deaths [6]. Pertussis is a class B reportable infectious disease in China (according to the epidemiological characteristics and damage degree, the law of the People’s Republic of China on the prevention and treatment of infectious diseases divides infectious diseases into three categories: A, B and C). According to data from the Chinese infectious disease reporting system, the incidence of pertussis has been less than 1 per 100 000 since the 1990s with only 2183 and 2517 pertussis cases being reported in 2011 and 2012, respectively [7]. In China, pertussis is mainly diagnosed by the physicians based on the typical clinical symptoms, as most of the Chinese hospitals do not have dedicated specific tests for pertussis(e.g. bacterial culture, serology test and molecular methods). Thus the reported low incidence may be related to the clinical and laboratory methods used for diagnosis, suggesting an underestimation of the disease prevalence.

In studies that have examined the prevalence of pertussis, most focused on children with a persistent cough for ≥2 weeks [8–10]. There is little data on the prevalence of pertussis in children with an unselected cough duration. Thus, in our prospective study, we sought to determine the prevalence of pertussis in Chinese children irrespective of the cough duration. Furthermore, as data examining the association between clinical features and cough duration of pertussis are still not available, we also explored the clinical characteristics of children with pertussis with different cough durations. Our study’s primary aim was to compare the similarities and differences in the clinical features, microbiology, CRP and peripheral white blood cell count indices among 312 children with acute, sub-acute or chronic cough, with and without pertussis. Our secondary aim was to examine the antimicrobial susceptibility of all the *B. pertussis* isolates. We hypothesize that pertussis can be found in cough of any duration and that there are distinguishing features among the groups.

## Methods

### Study design and participants

A prospective study was undertaken between January 1st, 2016 and May 31st, 2017 from the in- and out-patient departments of the Jiangxi Provincial Children’s Hospital. Inclusion criteria for enrolled cases were children with: (a) the presence of cough (acute defined as cough duration < 2 weeks, sub-acute 2–4 weeks, chronic as >4 weeks) [11], (b) aged ≤14 yrs., (c) with no fever and the absence of wheezing, (d) the absence of findings suggestive of an alternative etiology other than Pertussis for a cough (e.g. digital clubbing, as specified in chronic lung or cardiac disease; failure to thrive, as specified in immunodeficiency, cystic fibrosis). Exclusion criterion were: (a) those who had been previously enrolled into this study, or (b) known chronic lung diseases (e.g. asthma), or other diseases (e.g. gastroesophageal reflux, cardiac diseases or immunodeficiency).

Potential recruits were approached by the primary author, identified through clinical chart and physical examination within 48 h of admission or the first time visit. Those who fulfilled the inclusion criteria were provided the relevant information for recruitment. Upon informed consent, demographic data and a detailed medical history (including vaccination, DTaP was widely used after 2012 in China, normally, three doses of DTaP are routinely administrated to infants at 3, 4, and 5 months of age, and a booster dose is given at 18 mo of age) were obtained, and a clinical examination was performed. Bilateral deep posterior nasopharyngeal swabs (NPS) and venepuncture for full blood count, CRP and serology and sputum were obtained when possible. The study was approved by the Ethical Committee of Jiangxi Provincial Children’s Hospital and written informed consent was obtained from each parent/guardian.

### Sputum and microbiological identification

Spontaneous sputum specimens were collected if the child could expectorate and sputum induction was performed if the child could not expectorate. The sputum specimens were processed by the hospital’s microbiology lab within 30 min. Immunofluorescence was used to determine the presence of the following viruses in the sputum: respiratory syncytial virus (RSV), adenovirus, influenza (A and B) virus, and para-influenza (1,2,3) virus (Diagnostic Hybirds, Inc.).

The two NPS specimens through both sides of nasal cavity were processed for pertussis culture respectively and then combined for PCR. For the culture, each NPS was immediately plated onto one charcoal agar (OXOID, UK) plate supplemented with 10% defibrinated sheep blood and cephalexinand incubated at 35–37 °C. After the culture, the NPS was frozen at − 80 °C immediately. The agar plate was incubated for 7 days. Colonies suspected to be *B. pertussis* underwent the slide agglutination test with *B.pertussis* and *B.parapertussis* antisera (Remel Europe Ltd., UK) [12]. Confirmed colonies were sub-cultured on a new charcoal blood agar plate without cephalexin for preservation. All isolated strains were preserved at − 60°Cuntil further analysis.

For the PCR, a real-time PCR method that amplified the specific genome of *B. pertussis* DNA was used, i.e. the promoter region of the gene encoding the pertussis toxin S1 subunit (ptxA) and the insertion element IS481 [13], the sequences of ptxA-Pr were 5′ -CCA ACG CGCA TG CG TG CAG ATTCGTC-3′ and 5′ -CCCTCTG CG TTTTG ATGG TGCCTA TTTTA − 3′, the sequences of IS481 were 5′-G ATTCAA TAGGTTG TA TGCATGGTT-3′ and 5′ - TGG ACCATTTCGAG T CGACG-3′. The amplicons were 191 bp and 145 bp in size respectively. The real-time PCR result for *B. pertussis* positive was defined by a positive amplification signal in both the IS481 PCRs and ptxA-pr.

E-test strips were used for testing the susceptibility of the *B.pertussis* isolates against erythromycin, azithromycin, sulphamethoxazole/trimethoprim, levofloxacin, amoxicillin and doxycycline. The MICs determined by the E-test were measured after 4 days of incubation. *Staphylococcus aureus*atcc29213 were included in each batch of susceptibility tests for the quality control.

Because there was no breakpoints for *B.pertussis* colonizing recommended by the Clinical and Laboratory Standards Institute (CLSI) [14] and the European Committee on Antimicrobial Susceptibility Testing (EUCAST) [15], the MIC50, MIC90 and MIC ranges were reported in detail. The breakpoints of *Haemophilus influenza* colonizing usually in the nasopharynx in CLSI and EUCAST were borrowed to analyze the susceptibility of the present *B.pertussis* isolates. This analysis was to reveal the susceptibility in a familiar way.

### Blood and serology

Peripheral blood samples were examined for CRP, white cell count indices, IgM serology for *Mycoplasma pneumoniae* (MP)*, Chlamydia pneumoniae* (CP),Respiratory syncytial virus (RSV) and *Legionella pneumophilia* (LP) and IgG for anti-pertussis toxin (anti-PT) and anti-pertactin (anti-PRN). Commercially available ELISA kits (FUJIREBIO INC.; ZHU HAI LIVZON DIAGNOSTICS INC.; BEIJING BEIER BIOENGINEERING CO.LTD; Euroimmun, Lübeck, Germany) were used for all the serology. The pertussis antibody results were reported in international units per milliliter (IU/ml) and referred to the “First International Standard for Pertussis Antiserum”, NIBSC code: 06/140, World Health Organization (WHO).

### Pertussis case definition

A patient was considered to have definite *B. pertussis* infection if culture or PCR was positive for *B. pertussis* [16]. ‘Probable *B. pertussis* infection’ was defined when an anti-PT IgG was ≥62.5 IU/ml if the patient had not had the vaccination against pertussis in the previous 12 months [17]. Either definite pertussis or probable pertussis was considered as laboratory confirmed pertussis in this study.

### Statistical analysis

All analyses were performed using SAS v.10 (SAS Institute Inc., USA). Chi-squared test and Fisher’s exact test were used to compare the frequencies between the groups. Wilcoxon/Krusakal Wallis test was used to compare continuous variables among the groups. Where continuous data were not normally distributed, medians with accompanying interquartile ranges (IQR) are presented. Statistical significance was considered present if the two-tailed *p-value was* < 0.05.

## Results

Among 320 patients initially approached, eight were excluded because their parents either declined to participate or their specimens were inappropriate. Therefore, a total of 312 patients were finally included in this study. The median age of the 312 children enrolled was 3 months (IQR 3–34), ranging from 1 month to 11 years; 210 (67.3%) were male, and 142 (45.5%) were vaccinated against pertussis in the previous 12 months. Nasopharyngeal swabs were collected from 309 (99%) enrolled children for pertussis culture and PCR analysis. For peripheral blood samples, 298 (95.5%) were obtained for the examination of IgG for anti-pertussis toxin, 217 (69.6%) for anti-pertactin; 289 (92.6%) were obtained for the examination of IgM for *Mycoplasma pneumoniae* (MP)*, Chlamydia pneumoniae* (CP),Respiratory syncytial virus (RSV) and *Legionella pneumophilia* (LP); and 296 (94.9%) were obtained for full blood count and CRP test. Two hundred and fifty-four (81.4%) sputum samples were obtained for immunofluorescence.

Table 1 presents the prevalence of *B. pertussis* infection in children presenting with cough. Laboratory confirmed pertussis was most common in the sub-acute group compared to the acute group and chronic group. Anti-PT IgG ≥ 62.5 IU/ml presence was most common in the sub-acute compared to the acute group and chronic group.

**Table 1:** Prevalence of *B. pertussis* infection in children presenting with cough

	Acute cough *N* = 119	Sub-acute cough *N* = 80	Chronic cough *N* = 113	*P* value
Anti –PT IgG (*N* = 298), ≥ 62.5 IU/ml, *n*(%)	*N* = 117 14(12.0)	*N* = 73 28(38.4)	*N* = 108 32(30.0)	<0.0001
Anti –PRN IgG, IU/ml (*N* = 217)	*N* = 95	*N* = 57	*N* = 65	
median, IQR	11.1, 0–33.3	12.4, 0–48.0	22.9, 8.7–98.0	0.0082
NPS culture and PCR for pertussis (*N* = 309)	*N* = 119	*N* = 79	*N* = 111	
Culture positive, *n*(%)	11(9.2)	20(25.3)	5(4.5)	<0.0001
PCR positive, *n*(%)	25(21.0)	30(38.0)	14(12.6)	0.0002
	*N* = 119	*N* = 80	*N* = 113	
Laboratory confirmed pertussis, *n*(%)	34(28.6)	37(46.3)	26(23.0)	0.0021

The difference in clinical characteristics, microbiology, CRP and peripheral white cell count indices between children presenting with acute cough diagnosed with laboratory confirmed pertussis or not are presented in Table 2. Among the 119 children with acute cough, only two features were significantly different between the groups: apnea and cyanosis. Notably paroxysmal cough was very common in both groups (> 75%) and wet/productive cough was as likely found in the pertussis group as in those without pertussis. The proportion of other pathogens (any virus and *Mycoplasma pneumoniae, Chlamydia pneumoniae*, Respiratory syncytial virus and *Legionella pneumophilia*) detected between laboratory confirmed pertussis or not did not show significant differences. There was no significant difference of CRP and peripheral white cell count indices between children with and without laboratory confirmed pertussis.

**Table 2:** The differences of clinical characteristics, microbiology, CRP and peripheral white cell count indices between children presenting with acute cough diagnosed with or without laboratory-confirmed pertussis

	Laboratory confirmed Pertussis^a^		*P* value
	Yes *N* = 34	No *N* = 85	
Age group (months)			
0 - <12, *n*(%)	22(64.7)	61(71.8)	0.3491
12–36, *n*(%)	5(14.7)	15(17.7)	
36 +, *n*(%)	7(20.6)	9(10.6)	
Gender			
Male, *n*(%)	16(47.1)	62(72.9)	0.0073
Female, *n*(%)	18(52.9)	23(27.1)	
Contact with cough patient			
Yes, *n*(%)	20(58.8)	40(47.1)	0.2462
No, *n*(%)	14(41.2)	45(52.9)	
Clinical features			
Fever, *n*(%)	11(32.4)	32(37.7)	0.5871
Apnea, *n*(%)	6(17.7)	5(5.9)	0.0453
Cyanosis, *n*(%)	10(29.4)	9(10.6)	0.0113
Cough characteristic			
Whoop, *n*(%)	7(20.6)	10(11.8)	0.2140
Paroxysm, *n*(%)	28(82.4)	64(75.3)	0.4062
Wet/Productive, *n*(%)	16(47.1)	42(49.4)	0.8166
Nocturnal cough, *n*(%)	15(44.1)	31(36.5)	0.4390
Post-tussive vomiting, *n*(%)	9(26.5)	26(30.6)	0.6561
Other pathogens positive	*N* = 30	*N* = 72	
Immunofluorescence (*N* = 102)			
Any virus positive, *n*(%)	12(40.0)	33(45.8)	0.5888
IgM for MP*,* CP,RSV and LP (*N* = 110)	*N* = 32	*N* = 78	
MP - IgM positive, *n*(%)	0(0)	3(3.9)	0.2607
CP –IgM positive, *n*(%)	4(12.5)	3(3.9)	0.0913
RSV – IgM positive, *n*(%)	2(6.3)	2(2.6)	0.3483
LP – IgM positive, *n*(%)	4(12.5)	7(9.0)	0.5756
Peripheral white cell count indices and CRP (*N* = 115)	*N* = 34	*N* = 81	
Peripheral wbc count(median, IQR)	12.2(9.4–17.2)	10.5(8.0–14.9)	0.2673
Neutrophil absolute count(median, IQR)	3.1(2.3–5.4)	3.2(1.8–6.1)	0.9536
Lymphocyte absolute count(median, IQR)	7.4(3.3–12.8)	5.2(2.8–7.9)	0.0509
CRP(median, IQR)	1.6(0.1–6.1)	2.7(0.1–7.7)	0.4815

^a^Either definite pertussis or probable pertussis was considered as laboratory confirmed pertussis in this study

Table 3 shows the differences of clinical characteristics, microbiology, CRP and peripheral white cell count indices between children presenting with sub-acute cough diagnosed with laboratory confirmed pertussis or not. Among 80 enrolled children with sub-acute cough, whoop was the only featured symptom reported by children who had laboratory evidence of pertussis. Remarkably, paroxysmal cough was also very common in children with sub-acute cough either with or without laboratory evidence of pertussis (> 74%). The proportion of other pathogens (any virus and *Mycoplasma pneumoniae, Chlamydia pneumoniae*, *Respiratory syncytial virus* and *Legionella pneumophilia*) detected between children with laboratory confirmed pertussis or not did not show significant differences. There was no significant difference of CRP and peripheral white cell count indices between laboratory confirmed pertussis or not.

**Table 3:** The differences of clinical characteristics, microbiology, CRP and peripheral white cell count indices between children presenting with sub-acute cough diagnosed with or without laboratory-confirmed pertussis

	Laboratory confirmed pertussis^a^		*P* value
	Yes *N* = 37	No *N* = 43	
Age group (months)			
0 - <12, *n*(%)	29(78.4)	31(72.1)	0.1936
12–36, *n*(%)	7(18.9)	6(14.0)	
36 +, *n*(%)	1(2.7)	6(14.0)	
Gender			
Male, *n*(%)	25(67.6)	30(69.8)	0.8324
Female, *n*(%)	12(32.4)	13(30.2)	
Contact with cough patient			
Yes, *n*(%)	20(54.1)	29(67.4)	0.2204
No, *n*(%)	17(46.0)	14(32.6)	
Clinical features			
Fever, *n*(%)	5 (13.5)	17(39.5)	0.0094
Apnea, *n*(%)	7(18.9)	6(14.0)	0.5483
Cyanosis, *n*(%)	8(21.6)	7(16.3)	0.5416
Cough characteristic			
Whoop, *n*(%)	20(54.1)	9(20.9)	0.0021
Paroxysm, *n*(%)	32 (86.5)	32 (74.4)	0.1785
Wet/Productive, *n*(%)	20(54.1)	18 (41.9)	0.2762
Nocturnal cough, *n*(%)	25(67.6)	21 (48.8)	0.0911
Post-tussive vomiting, *n*(%)	18 (48.7)	24 (55.8)	0.5223
Other pathogen positive			
Immunofluorescence (*N* = 73)	*N* = 34	*N* = 39	
Any virus positive, *n*(%)	12 (35.3)	10 (25.6)	0.3699
IgM for MP*,* CP,RSV and LP (*N* = 73)	*N* = 33	*N* = 40	
MP - IgM positive, *n*(%)	0(0)	0(0)	–
CP –IgM positive, *n*(%)	0(0)	2 (5.0)	0.1927
RSV – IgM positive, *n*(%)	1 (3.0)	1(2.5)	0.8901
LP – IgM positive, *n*(%)	0(0)	4 (10.0)	0.0617
Peripheral white cell count indices and CRP (*N* = 76)	*N* = 32	*N* = 42	
Peripheral wbc count(median, IQR)	11.2 (8.4–16.5)	10.5(8.2–14.0)	0.4918
Neutrophil absolute count(median, IQR)	2.6 (1.8–3.5)	2.2(1.4–3.8)	0.4884
Lymphocyte absolute count(median, IQR)	7.6 (4.7–10.9)	7.3(3.7–9.5)	0.3342
CRP(median, IQR)	0.1 (0.1–3.8)	0.1 (0.1–4.5)	0.8594

^a^Either definite pertussis or probable pertussis was considered as laboratory confirmed pertussis in this study

The differences of clinical characteristics, microbiology, CRP and peripheral white cell count indices between children presenting with chronic cough diagnosed with laboratory confirmed pertussis or not are presented in Table 4. No statistical significance in “classic” clinical pertussis symptoms was observed between children with and without laboratory evidence of pertussis. The proportion of other pathogens (any virus and *Mycoplasma pneumoniae, Chlamydia pneumoniae*, *Respiratory syncytial virus* and *Legionella pneumophilia*) detected between laboratory confirmed pertussis or not did not show significant difference. There was no significant difference of CRP and peripheral white cell count indices between laboratory confirmed pertussis or not.

**Table 4:** The differences of clinical characteristics, microbiology, CRP and peripheral white cell count indices between children presenting with chronic cough diagnosed with or without laboratory-confirmed pertussis

	Laboratory confirmed pertussis^a^		*P* value
	Yes *N* = 26	No *N* = 87	
Age group (months)			
0 - <12, *n*(%)	14 (53.9)	27 (31.0)	0.0686
12–36, *n*(%)	2 (7.7)	19 (21.8)	
36 +, *n*(%)	10 (38.5)	41 (47.1)	
Gender			
Male, *n*(%)	17 (65.4)	60 (69.0)	0.7310
Female, *n*(%)	9 (34.6)	27 (31.0)	
Contact with cough patient			
Yes, *n*(%)	14 (53.9)	39 (44.8)	0.4188
No, *n*(%)	12 (46.2)	48 (55.2)	
Clinical features			
Fever, *n*(%)	11 (42.3)	39 (44.8)	0.8204
Apnea, *n*(%)	2 (7.7)	2(2.3)	0.1916
Cyanosis, *n*(%)	3 (11.5)	3(3.5)	0.1065
Cough characteristic			
Whoop, *n*(%)	8 (30.8)	20 (23.0)	0.4200
Paroxysm, *n*(%)	17 (65.4)	38 (43.7)	0.0520
Wet/Productive, *n*(%)	12 (46.2)	31 (35.6)	0.3323
Nocturnal cough, *n*(%)	6 (23.1)	22 (25.3)	0.8188
Post-tussive vomiting, *n*(%)	9 (34.6)	28 (32.2)	0.8167
Other pathogen positive			
Immunofluorescence (*N* = 79)	*N* = 17	*N* = 62	
Any virus positive, *n*(%)	6 (35.3)	9 (14.5)	0.0530
IgM for MP*,* CP,RSV and LP (*N* = 106)	*N* = 25	*N* = 81	
MP - IgM positive, *n*(%)	1 (4.0)	7 (8.6)	0.4424
CP –IgM positive, *n*(%)	0(0)	2(2.5)	0.4277
RSV – IgM positive, *n*(%)	1 (4.0)	7 (8.6)	0.4424
LP – IgM positive, *n*(%)	0(0)	8 (9.9)	0.1022
Peripheral white cell count indices and CRP (*N* = 107)	*N* = 25	*N* = 82	
Peripheral wbc count(median, IQR)	9.0 (6.3–15.6)	9.1 (7.2–12.3)	0.7600
Neutrophil absolute count(median, IQR)	3.1 (1.8–4.5)	3.5 (2.2–4.9)	0.2344
Lymphocyte absolute count(median, IQR)	4.3 (2.7–8.9)	3.9 (2.6–6.4)	0.6454
CRP(median, IQR)	1.8 (0.3–4.6)	1.4 (0.1–6.6)	0.7982

^a^Either definite pertussis or probable pertussis was considered as laboratory confirmed pertussis in this study

The MIC50, MIC90 and MIC range values of all the 36 *B.pertussis* isolates to 6 antimicrobials were shown in Table 5. In the test, 72.2% (26/36) of the 36 isolates could not be inhibited by erythromycin and azithromycin at all, and the remaining 10 isolates (27.8%) demonstrated low MICs, and were susceptible to erythromycin and azithromycin interpreted by the breakpoints for *H.influenzae*. No MIC of *B.pertussis* isolates to sulphamethoxazole/trimethoprim was more than 1.5 mg/L in the test. The MIC to levofloxacin was no more than 1.5 mg/L in the test. The MIC50 and MIC90 to amoxicillin were 0.75 mg/L and 1 mg/L respectively, and the tested isolates were sensitive to amoxicillin by the EUCAST points. Doxycycline did not show potent action on these isolates in in-vitro test.

**Table 5:** Antimicrobial susceptibility test results of the 36 *B. pertussis* isolates

Antimicrobials	MICs (mg/L)			S% by breakpoints for *H.influenzae*	
	MIC 50	MIC 90	MIC range	in CLSI	in EUCAST
erythromycin	> 256	> 256	0.064 - > 256	–	27.8
azithromycin	> 256	> 256	0.047 - > 256	27.8	27.8
sulphamethoxazole/ trimethoprim	0.125	0.38	0.023–1.5	88.9	88.9
levofloxacin	0.75	1	0.5–1.5	100	97.2
amoxicillin	0.75	1	0.5–2	–	100
doxycycline	4	6	2–8	–	0

## Discussion

We investigated the prevalence and clinical characteristics, CRP and peripheral white cell count indices of pertussis in children irrespective of the cough duration. In 312 children with cough presenting to the in- and out-patient departments of one large Chinese hospital, 97 (31.1%) children had laboratory evidence of pertussis. Although confirmed pertussis was found in all cough duration groups (acute, sub-acute and chronic), it was significantly higher in those with sub-acute cough. Among those acute cough, apnea and cyanosis were significantly more common in those with pertussis; whilst in children with sub-acute cough, whoop was the sole statistically significant factor. In the chronic cough group, none of these factors were significant between groups.

There is little data on the prevalence of pertussis in children based on cough duration. We found laboratory confirmed pertussis in all the different cough duration groups, but significantly higher in the sub-acute group than in the acute or chronic groups. Although the classic symptoms of pertussis were more common in the lab-confirmed pertussis group, most of these features were also present in the children without pertussis. A multicenter study of epidemiological data of pertussis infection among adolescents and adults with a cough of a duration≤30 days reported 6.9% of enrolled patients had pertussis infection. The classic symptoms of pertussis were present in confirmed cases or not [18], but the investigation of the incidence rate of pertussis was based on age groups, and the cases with persistent and chronic cough were not included in this study. A New Zealand prospective study of school-age children (aged 5–16 years) and adults (17–49 years) with cough of 2 weeks duration or greater reported 10% (23/225) had pertussis infection and neither cough duration nor any individual presenting symptom discriminated those with pertussis from those due to other causes [19]. The prevalence of pertussis was based on age groups and cases with cough of less than 2 weeks were not included in this study.

There is little published data that has described the clinical characteristics in children with pertussis of various cough duration. We found that apnea and cyanosis were more common in the acute cough group, whilst whoop was more common in the sub-acute cough group. However, we did not find any specific characteristic in the chronic cough group. This highlights that a “one-size-fits-all” clinical case definition is not appropriate as also described by several others [16]. Indeed, a growing body of evidence suggests that the existing WHO clinical case definitions of pertussis(a case diagnosed as pertussis by a physician, or a person with a cough lasting ≥2 weeks with ≥1 of the following symptoms: paroxysms (ie, fits) of coughing, inspiratory “whooping”, and posttussive vomiting (ie, vomiting immediately after coughing) without other apparent cause [16]) may be not appropriate anymore.

The spectrum of illness for pertussis can vary widely with atypical pertussis commonly found in adolescents, adults, and in infants under 1 year of age [20, 21]. A small study of 76 adult patients with cough of more than 2 weeks found 14 patients (18.4%) had laboratory evidence of pertussis infection, and classic symptoms of pertussis were not significantly common in confirmed patients [22]. A case-control study in children0 to 18 years of age found only 42% of confirmed cases met a clinical definition of pertussis and typical symptoms were not common in confirmed cases, and the atypical symptoms were likely moderated by high immunization rates or may have been caused by other respiratory pathogens [23]. A small study of 7 neonates with confirmed pertussis by bacterial culture reported cyanosis and apnea were the characteristic symptoms displayed after 4–7 days of the onset [24].These findings may reflect that ‘classic’ symptoms of pertussis may not necessarily be evident, and that clinical symptomatology is influenced by patient age, immunization status and underlying co-morbidities, but the clinical characteristics of patients with pertussis of different cough durations were unreported in these studies. With consideration of patient age, immunization status and underlying co-morbidities, our study found clinical symptomatology of pertussis was associated with cough duration. Our results raise questions regarding the possible need to reexamine the existing diagnostic criteria for pertussis, and possibly identify new sets of criteria specific to particular subgroups based on cough duration.

In our study, other than culture and PCR, we detected IgG for both anti-PT and anti-PRN. Pertussis toxin is secreted exclusively by *B. pertussis*, whereas it is now clear that antibody response to pertactin also occurs following other *Bordetella* infections, so that isolated increases in titers of antibody against pertactin are not specific for *B. pertussis* infection. For this reason, the measurement of anti-PT antibodies is recommended [5]. In our study, a child was considered to have probable B. pertussis infection when an anti-PT IgG ≥ 62·5 IU/ml if the patient had not had the vaccination against pertussis in the previous 12 months [17].

In the 36 *B.pertussis*isolates detected, macrolide resistance with MIC > 256 mg/L was high, consistent with a previous study in China [12].Currently, marolides are recommended as first line antibiotics for the treatment of pertussis [25, 26] and sulphamethoxazole/trimethoprim as the second choice [27–29]. Our results suggest that amoxicillin maybe an alternative for children with marolide-resistant *B.pertussis* infection and that local sensitivities are required to inform clinical practice.

A strength of our study is the detailed co-detection of other pathogens that may exist with pertussis. Our study has many limitations. Firstly, we did not analyze how co-infection of other pathogens influence the cough symptoms. Secondly, we limited children enrolled under 14 years old, and we did not recruit children with alternative etiologies (e.g. cystic fibrosis, immune deficiencies), because these children might also get pertussis. Thirdly, we did not do chest X-ray interpretation/comparisons and not conduct a specific age breakdown for apnea and cyanosis because of small sample size. Fourthly, we did not have data from asymptomatic healthy controls. Further our study was limited to a single-center study and may also not be representative of all children.

## Conclusions

Several findings of the current study were important for clinical practice: (a) laboratory confirmed pertussis can be present in children irrespective of cough duration; (b) children with pertussis with different cough durations have clinical features that are influenced by the child’s age and immunization state; and(c) those with chronic cough have nonspecific cough, lacking characteristic symptoms. This may suggest that in the context of the likely underestimation of the burden of pertussis, the “one-size-fits-all” clinical pertussis case definition is no longer optimal to recognize this disease in a population and large comprehensive studies of children with all types of cough is required to confirm or refute our findings. This may make substantial inroads into increasing both the sensitivity and specificity in pertussis diagnosis, will impact public health.

This study also suggests that amoxicillin maybe an alternative for children with marolide-resistant *B.pertussis* infection. But these analysis were only based on the in vitro test, as the dose and course of treatment were not clear. Clinical studies should be designed and performed to confirm that this alternative drug could eliminate the bacteria in vivo effectively.

## Data Availability

All data generated or analysed during this study are included in this published article.

## Abbreviations

*B.*: *Bordetella pertussis*
CLSI: Clinical and Laboratory Standards Institute
CP: *Chlamydia pneumoniae*
EUCAST: European Committee on Antimicrobial Susceptibility Testing
IQR: Interquartile ranges
LP: *Legionella pneumophilia*
MP: *Mycoplasma pneumoniae*
NPS: Nasopharyngeal swabs
PRN: Pertactin
PT: Pertussis toxin
RSV: Respiratory syncytial virus
